# Stakeholder Engagement in Adoption, Implementation, and Sustainment of an Evidence-Based Intervention to Increase Mammography Adherence Among Low-Income Women

**DOI:** 10.1007/s13187-021-01988-2

**Published:** 2021-03-22

**Authors:** Jennifer Holcomb, Gayla M. Ferguson, Jiali Sun, Gretchen H. Walton, Linda Highfield

**Affiliations:** 1 Department of Management, Policy and Community Health, The University of Texas Health Science Center at Houston (UTHealth) School of Public Health, Houston, TX USA; 2 Department of Epidemiology, Human Genetics and Environmental Sciences, The University of Texas Health Science Center at Houston (UTHealth) School of Public Health, Houston, TX USA; 3 Department of Internal Medicine, The University of Texas Health Science Center at Houston (UTHealth) John P and Katherine G McGovern Medical School, Houston, TX USA

**Keywords:** Stakeholder engagement, Consolidated Framework for Implementation Research, Mammography, Evidence-based intervention, Community-based participatory research

## Abstract

Multi-level organizational stakeholder engagement plays an important role across the research process in a clinical setting. Stakeholders provide organizational specific adaptions in evidence-based interventions to ensure effective adoption, implementation, and sustainability. Stakeholder engagement strategies involve building mutual trust, providing clear communication, and seeking feedback. Using constructs from the Consolidated Framework for Implementation Research and The International Association for Public Participation spectrum, a conceptual framework was created to guide stakeholder engagement in an evidence-based intervention to increase mammography appointment adherence in underserved and low-income women. A document review was used to explore the alignment of the conceptual framework with intervention activities and stakeholder engagement strategies. The results indicate an alignment with the conceptual framework constructs and a real-world application of stakeholder engagement in a mammography evidence-based intervention. The conceptual framework and stakeholder engagement strategies can be applied across a range of community-based cancer programs and interventions, organizations, and clinical settings.

## Background

With renewed interest in patient-centered care, stakeholder engagement (SE) is in the spotlight [1–4]. SE is a bidirectional partnership between researchers with patients, clinical and community partners, and other healthcare stakeholders to achieve a desired outcome [1, 3, 5]. Stakeholders can be engaged in a range of research activities including planning, proposal development, data collection, data analysis, and dissemination of results [3, 6]. Previous research has shown infrequent SE with clinical stakeholders who are not clinicians [1]. SE is more common in early research stages when topic planning is pursued, and in contrast to implementation and dissemination stages of research [1]. A transition step of practical trainings and tools for advancing SE within various research stages has shown to be limited to date [1]. Further examination is necessary to define the extent of SE across the research process*. The International Association for Public Participation* (IAP2) spectrum is a framework for SE across multiple research stages [5, 7, 8]. The IAP2 framework outlines five participation phases hypothesized to influence SE and decision-making: *Inform*, *Consult*, *Involve*, *Collaborate*, and *Empower* [5, 8]. Implementation science is then helpful to identify specific mechanisms to successfully engage multiple stakeholders within each phase. *The Consolidated Framework for Implementation Research* (CFIR) is a comprehensive conceptual framework composed of 39 constructs associated with effective intervention implementation identified in implementation science theories and empirical studies [9, 10]. The constructs are organized by five major domains: *Intervention Characteristics*, *Inner Setting*, *Outer Setting*, *Characteristics of individuals involved in implementation*, and *the Implementation Process* [9]. CFIR provides a pragmatic structure for engaging multiple stakeholders to promote effective program planning and implementation in evidence-based interventions (EBIs) [9–11].

Although there is an increasing focus on stakeholder-engaged studies, more research is needed to better understand the role of SE in breast cancer research [12]. Previous research to improve breast cancer screening suggests the need to implement patient-centered tools to relay technical and process knowledge to women seeking a mammogram [13, 14]. To ensure effectiveness of these tools and stakeholder capacity to utilize these tools, stakeholders need to be engaged in mammography screening intervention implementation and dissemination [13, 14]. A research to practice gap exists in understanding how to effectively engage stakeholders in EBIs to improve mammography screening adherence. Few studies examining how to effectively adapt and scale mammography screening EBIs within a specific setting even exist [15, 16]. SE is a key in the dissemination and implementation of breast cancer EBIs across all research stages [17]. Multi-level SE can provide site specific considerations for effective adaptation of EBIs into practice [17]. The Patient-Centered Outcomes Research Institute (PCORI) published guidelines for patient and SE in breast cancer research which included the themes: *Authenticity*, *Real-World Perspective*, *Mutual Trust*, *Plain Language*, *Equitable Partnerships*, *Relationship Building*, *Community Engagement*, and *Feedback* [12]. Real-world application of the SE guidelines is needed to develop effective, appropriate programs and robust research results to impact disparities in breast cancer diagnosis and screening [12].

## Peace of Mind Program

Breast cancer is the second leading cause of death in US women, with the highest mortality rates seen among Latino and African American women [18, 19]. While mammogram screening and early diagnosis reduce breast cancer morbidity and mortality, racial and economic disparities in mammography screening access and adherence continue to exist, leading to delayed diagnosis and worsened prognosis [20, 21]. The goal of the Peace of Mind Program (PMP) was to bridge the research to practice gap through the dissemination and implementation of a multi-level EBI to increase mammography appointment adherence in underserved women [16, 22]. The program was a telephone-based reminder phone call intervention to assess a woman’s readiness to attend a mammogram appointment and provide structured counseling for both cognitive and system barriers (e.g., paperwork and cost). A full description of PMP has been reported elsewhere [16, 22]. In EBI development, interviews and focus groups to examine barriers and facilitators of mammography screening were conducted with African American women whose clinical partners identified them as having missed a mammography appointment within the last 6 months [23]. Similar patient stakeholders reviewed and provided feedback on scripts for a previously adapted PMP program for underserved women. With clinical and organizational level stakeholders, intervention mapping (IM) was used to supplement the current structure and to develop an implementation intervention to facilitate the program adoption, implementation, and maintenance in federally qualified health centers (FQHCs) and charity clinics [15, 16]. The IM process incorporates theory and evidence-based health promotion planning and engaging multi-level stakeholders throughout the planning and implementation process [24]. IM methods have been used to guide the design and implementation of EBIs, but the application of IM in the PMP was an innovative approach to expansion and adaption of a mammography screening intervention in FQHCs and charity clinics [16]. A planning group was created to guide program intervention planning and implementation [16]. The planning group included researchers and administrative staff and certified community health workers (CHWs) from The Breast Health Collaborative of Texas (BHCTexas)—a nonprofit organization focused on ending breast cancer disparities with experience working on mammography screening programs in FQHCs and charity clinics. The planning group identified three levels of stakeholders at FQHCs and charity clinics across the BHCTexas membership network which included *Decision-Makers*, *Program Champions*, and *Patient Navigators*. *Decision-Makers* made critical decisions related to participation in PMP and implementation changes needed in their clinic. The *Decision-Makers* identified a *Program Champion* to be the key contact person for PMP in their clinic. The *Program Champion’s* primary responsibility was to help facilitate implementation at the clinic, promote PMP, and support new adopters. *Patient Navigators*, all of who were CHWs, worked directly with patients to schedule mammogram appointments, provide reminder calls, and obtain patient consent for inclusion in the intervention.

Utilizing SE principles and CFIR constructs, we developed a conceptual framework to reflect SE in PMP. We compared our conceptual framework with the stakeholders’ experiences and a review of program documents. We describe the stakeholders targeted and SE strategies across program activities. We then aimed to identify SE barriers and facilitators with a focus on the readiness of the stakeholders to promote adoption, implementation, and sustainment of PMP.

## Methods

PMP was implemented in the Greater Houston area in 25 clinical delivery locations; three mobile mammography providers and sixteen FQHCs and safety-net clinics. A grouped stepped-wedge design was used with three groups of clinics in each wedge moving from baseline to intervention at pre-set time periods [22]. Each group was made up of clinic *Decision-Makers*, *Program Champions*, and *Patient Navigators* from each clinic. PMP implementation included an adoption meeting to recruit each clinic for PMP. A site assessment meeting followed the adoption meeting at each clinical location to determine current clinic mammogram program processes and capacity for PMP implementation. Each group of clinics participated in nine stakeholder committee meetings, trainings on EBI methods and research ethics, and received ongoing support from the BHCTexas CHWs and researchers. The stakeholder committee meetings focused on the review of program adaptation and implementation materials; the use of program scripts; stakeholder assessment of implementation readiness; stakeholder discussion of program adaptation recommendations; and implementation problem solving.

### Conceptual Framework for Stakeholder Engagement

**Fig. 1:**
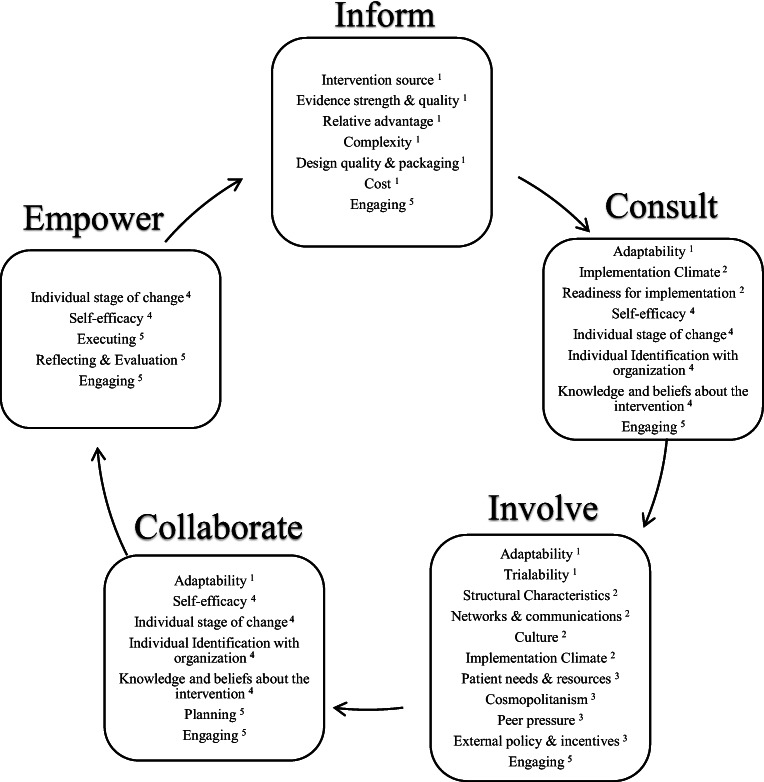
Combined conceptual framework for stakeholder engagement in the Peace of Mind Program (PMP). Adapted from International Association of Public Participation (IAP2) framework and constructs from the Consolidated Framework for Implementation Research (CFIR) [5, 8, 9]. Consolidated Framework for Implementation Research (CFIR) Domains indicated in each International Association of Public Participation (IAP2) phase: Intervention Characteristics^1^; Inner Setting^2^; Outer Setting^3^; Characteristics of individuals involved in intervention implementation^4^; and Implementation Process^5^. Description: Black and white graphic with a continuing sequence of International Association of Public Participation (IAP2) phases in a circular flow with indicated Consolidated Framework for Implementation Research (CFIR) constructs and domains for each phase

As shown in Fig. 1, the five IAP2 phases outlined in the IAP2 framework combined with constructs from CFIR were used to guide SE in PMP [8, 9]. The *Inform* phase is an opportunity for researchers to teach the stakeholders about the intervention and encourage recruitment. Providing intervention source information, evidence of strength and quality, and relative advantage of the intervention helps improve stakeholders’ knowledge regarding the intervention. Addressing the complexity, design quality, packaging, and cost helps the stakeholders make an informed decision about their desire to participate. The researchers sought feedback from the stakeholders regarding implementation during the *Consult* phase. The constructs of adaptability, trialability, patient needs and resources, cosmopolitanism (i.e., the organization’s network with other organizations), implementation climate, culture, and structural characteristics were used to determine the methods of implementation. The stakeholders provide valuable information regarding external policy and incentives, peer pressure, networks, and communication styles. These constructs showed the organizational factors which would facilitate or hinder successful implementation. Once stakeholders provided their feedback regarding the barriers and facilitators for implementation, work continued in the *Involve* phase to ensure the plans and actions to leverage facilitators and overcome barriers for implementation were effective and accurately reflected the information provided by the stakeholders. Implementation climate, readiness, and adaptability were explored for each participating clinical site. The individual stages of change, self-efficacy, individual identification with organization, knowledge, and beliefs about the intervention of the clinic staff were explored. Throughout the *Collaborate* phase, researchers sought feedback from stakeholders on implementation struggles and successes and provided positive reinforcement, as well as suggestions for improvements. Throughout the *Empower* phase, researchers aimed to equip the stakeholders with skills and the resources for intervention sustainment. The conclusory goal of PMP was for stakeholders to continue the intervention activities, and use the tools to implement additional organizational changes. In the *Empower* phase, stakeholders gained confidence by addressing self-efficacy, individual stage of change, executing, reflecting, and evaluation.

### Research on Stakeholder Engagement

A qualitative research design was used to conceptualize the framework by examining PMP stakeholder experiences and program documents. Documents including program published articles, records, and adaptation and implementation materials were reviewed. The program records included the stakeholder enrollment letter; PMP introduction webinar; stakeholder committee meeting agendas, minutes, and sign-in forms available for each meeting; PowerPoint presentations reflecting meeting content available for each meeting; and PowerPoint presentations of stakeholder training on EBI methods and research ethics. The program materials included a *Stakeholder Committee Handbook*, *Implementation Guide*, and the *Clinic Handbook* included in the *Stakeholder Handbook*. A printed version of the *Stakeholder Handbook* what was provided to stakeholders through an online application. However, it was determined a printed version of materials should be provided as a back-up plan, and for those who prefer to use “flip-able” notebook pages. The *Stakeholder Committee Handbook* provided a PMP overview and committee onboarding process. The *Implementation Guide* contained instructions for clinical sites to implement PMP and templates for required staff, scheduling appointments at clinics, and site assessment and implementation readiness checklists. This guide provided an overview of the program delivery, administration, and evaluation. The *Clinic Handbook* provided each clinical implementation site with a digestible introduction to the program components and timeline. It contained job descriptions for the clinic staff involved in the program, an overview of the communication techniques used, barrier scripts for patient interactions, cumulative instructional Power Point slide sets, and approved informed consent forms.

A thematic content analysis was used to code and analyze SE document data [25, 26]. Coding began using an a priori set of codes from the conceptual framework based in SE and implementation science literature with program activities. Open coding was used to identify any codes not initially identified from these two sources. Coding included words and phrases used by stakeholders and tasks outlined in the documents. Two researchers initially identified 73 codes reflecting SE strategies within each PMP activity and IAP2 phase. The final 40 codes were considered in terms of their relationships to one another and were interpreted based on the corresponding 25 CFIR constructs within the five IAP2 phases in the conceptual framework. The complete code list was also interpreted in terms of facilitators and barriers in SE with the corresponding 25 CFIR constructs. Quality checks on identified codes were conducted by two additional researchers who did not participate in the initial coding. The researchers reviewed codes from each of the five corresponding IAP2 phases and double-checked their relevance and corresponding CFIR construct. Any discrepancies were resolved through iterative document review and discussion until consensus was reached.

## Results

### Stakeholder Engagement Strategies

Documents were mapped to identify SE strategies in each program activity corresponding with an IAP2 phase in the conceptual framework (Table 1). Building mutual trust, providing clear communication, and seeking feedback through SE early in program adoption were important for effective program implementation. The engagement strategies in the *Inform* phase focused on gaining buy-in from clinic decision-makers based on the Intervention Characteristics. Clinic *Decision-Makers* were engaged by email and participated in an initial adoption meeting and webinar with the researchers. The researchers provided information on the effectiveness of PMP to increase mammogram appointment adherence, the advantage of PMP compared to current practices and other mammography programs, the cost of PMP implementation, and the implementation plan and materials. Once a clinic agreed to participate, the researchers facilitated relationships with mobile mammography providers, if needed, for clinics to implement PMP. SE provided encouragement for clinics to adopt the program, cultivate awareness, and promote buy-in from the clinic *Decision-Makers* for continued participation in PMP. In the *Consult* phase, feedback on the PMP implementation was sought in the clinical site assessment meeting, stakeholder committee meetings, and mammography program drives based on the PMP Intervention Characteristics, Inner Setting of the clinic, and Individual Characteristics of the stakeholders participating in PMP. The climate of the clinic, readiness to participate in PMP, and adaptability of PMP to baseline practices were assessed by the clinic *Decision-Makers* and *Program Champions* were through a site assessment checklist. In the *Involve* phase, feedback about PMP in stakeholder committee meetings was used for PMP implementation planning and preparation. During the stakeholder committee meetings, mammography program drives for each clinic were reviewed and analyzed to provide baseline data and processes prior to training and implementation. The Inner Setting and Outer Setting characteristics in terms of the clinic’s relationships with other clinics providing competing services, state and local policies guiding the clinic, and the needs of the populations served were examined to adapt and refine PMP materials. These materials were then used during the trainings to ensure continuity and fidelity of implementation. After the trainings were conducted, an implementation readiness checklist was completed by each participating clinic to assist in initial implementation.

**Table 1:** Stakeholder engagement strategies in the Peace of Mind Program (PMP) activities across the International Association for Public Participation (IAP2) framework [8]

PMP Program Implementation		IAP2 Phases				
Activities	Stakeholders	Inform	Consult	Involve	Collaborate	Empower
Initial emails, webinar, and adoption meetings	Clinic decision makers	Invite clinics to participate in PMP Explain EBIs in breast cancer prevention Explain PMP participation expectations, objectives, and implementation	Obtain agreement to participate in PMP (MOUs) Facilitate relationships with mobile mammography providers, as needed Complete internal clinic paperwork as requested such as HIPAA forms, ability to access patient records, etc.			
Clinical site assessment meeting	Clinic decision makers Clinic program champion		Assess baseline processes for implementing PMP Get feedback on PMP “fit” with clinic and current processes			
Stakeholder Committee Meetings	Clinic decision makers Clinic program champions Clinic patient navigators		Establish stakeholder committee members, roles, vision, and norms, for clinics participating in PMP Review pre-program baseline data, processes, and lessons learned Review PMP objectives and implementation materials	Facilitate pre-program baseline data collection for mammography program Synchronize PMP objectives with the clinics and populations served Adapt PMP implementation and materials Assist clinic with readiness, assessment, preparation, and implementation roll-out	Facilitate problem solving surrounding barriers to PMP implementation Discuss dissemination of findings Develop e-newsletter to highlight challenges and patient stories (Re-enforcement/Role-modeling)	
Mammography program drives (during Stakeholder Committee Meetings)	Clinic decision makers Mobile mammography providers		Collect pre- program baseline data through survey Assist clinic staff and mammography providers in management of mammography program drives			
PMP Trainings (during Stakeholder Committee Meetings)	Clinic patient navigators BHCTexas patient navigators (CHWs)			Provide 2-day (8 hour) training on adapted PMP implementation and materials Provide training on ethical approval and protection of human subjects Provided continuing education unit (CEU) for certified community health workers	Provide PMP follow-up training, if needed	
Ongoing Support (Phone, Email, Face to face meetings, and onsite)	Clinic decision makers Clinic program champion Clinic patient navigators				Provide clinic onsite role modeling and support from BHCTexas navigators (CHWs) Provide technical assistance Facilitate program evaluation and budget impact analysis	Align ownership of PMP implementation and operations Coordinate program maintenance and wrap-up

PMP implementation began with the *Collaborate* phase and continued throughout the *Empower* phase. In the *Collaborate* phase, adaptability of PMP was continuingly explored through identifying implementation barriers and facilitating solutions. Stakeholder’s knowledge and beliefs, self-efficacy, individual stage of change to surrounding PMP implementation, and the stakeholder’s perception of their clinic site were examined through ongoing support from the researchers and BHCTexas. In the *Empower* phase, stakeholders executed PMP implementation on their own. The stakeholders were empowered to assess their own self-efficacy and individual stage of change, to evaluate and maintain intervention activities, and to address any implementation barriers.

### Barriers and Facilitators in Stakeholder Engagement

Several barriers in establishing or maintaining SE were identified alongside facilitators that enabled or promoted SE in each IAP2 phase. First, gaining initial buy-in of the clinic leadership (*Decision-Makers*) in the *Inform* phase at each clinical site was important for recruitment of clinic staff (*Program Champions* and *Patient Navigators*) and attendance at stakeholder committee meetings. In the few clinics without clinic leadership buy-in of PMP effectiveness and advantage, it proved more challenging to gain the clinic staff buy-in needed for valuable participation in the stakeholder committee meetings. For other clinics with clinic leadership buy-in, staff engagement created a climate that was more conducive for PMP success. Clinic staff aligned with the climate of the clinic and goals of PMP, which lead to readiness to participate in the program. The leadership set both the tone and the priorities for the clinic staff and could therefore assist or hinder the issues faced by the clinic staff including competing demands and meeting time availability. Second, in the *Consult* phase, readiness for implementation posed the biggest barrier in the lack of dedicated mammography program staff and office space at each clinical site location. The lack of dedicated program staff in some clinics made the engagement and requirement of dedicated stakeholder committee members to represent each role in the program a challenge. Successfully contacting clinic staff was contingent on the researchers having current contact information and maintaining an accurate contact information registry. Next, in the *Involve* phase, once all of the appropriate program staff from each clinic were identified for stakeholder meetings, Inner Setting characteristics rather than Intervention Characteristics or Outer Setting characteristics proved to be barriers to SE. Structural characteristics in the competing departments and various levels of staff hierarchy posed a challenge to the program goal of free and open communication during the stakeholder committee meetings. The culture of each clinic, first determined by the clinic leadership, and perceived by the clinic staff determined the scope and depth of communication and problem solving. The freedom with which a clinic staff would speak frankly about the implementation practices and/or concerns depended on the clinic’s culture and learning climate. This was particularly true for clinics with leadership active in the stakeholder process. The clinic’s staff needed to feel he or she possessed the autonomy to honestly discuss the intervention and happenings of the clinic without reprimand or negative consequences from leadership. The researchers observed clinic staff were hesitant to share struggles or information which could paint their respective organizations in a negative light. Though clinic staff were observed to be hesitant to describe potentially unflattering details about their program implementation to one another during stakeholder committee meetings, the staff did build a relationship and trust with researchers over time to share their struggles and concerns. Online communication and engagement seemed to provide the most flexibility for clinic staff and other stakeholders. Individual conference calls through UberConference software allowed each clinical site stakeholder to feel more comfortable sharing successes and problems with the implementation of the program at their site. While the UberConference software allowed for flexibility of scheduling, finding a consistent time that multiple individuals were available to meet was still a challenge. Adding scheduling to the competing demands of a clinic, many of which superseded the stakeholder committee meeting, made it difficult to find a consistent block of time to have all committee members or clinics present for the same meeting. The clinic staff performed multiple jobs and the demands on their time and attention left them distracted and at times, disinterested.

Barriers in program uptake and staff turnover were identified across IAP2 phases. Maintaining accurate contact information proved difficult with high staff turnover at participating clinical sites. In the *Consult* and *Involve* phases, staff turnover and competing demands also made it difficult to have consistent stakeholder committee meeting attendance. To identify and address any issues with differing program uptake and staff turnover, adapted program materials, trainings, and ongoing support provided in various modalities were provided. The program materials and trainings were adapted, packaged, printed, and distributed to the clinics’ staff. All methods were adapted to allow implementers to utilize the most effective delivery methods which involved stakeholder’s collaboration and feedback. The inclusion of BHCTexas CHWs in training and the opportunity to re-train staff as needed also added value to the self-efficacy of stakeholders and built staff capacity to implement the program. In the *Collaborate* phase, clinical staff counseling and peer support were a benefit of the stakeholder process. The CHWs collaborated and empowered the clinical staff to implement the program. The CHWs were embedded on site in the clinics which allowed for relationship and trust building to occur. By having CHWs that were familiar with the PMP practices, as well as completed their certification education and training, the clinical staff had individuals they could communicate with, model, and seek assistance from during trainings and implementation. Lastly in the *Empower* phase, the researchers and BHCTexas worked closely with clinic staff to transition program ownership and maintenance. Embedded in the clinic at the beginning of implementation, the CHWs role modeled the reminder phone call process and over time integrated clinic staff into leading the process. The CHWs provided reinforcement through reflection and evaluation to improve self-efficacy of clinic staff. The goal was to have clinic staff take ownership in executing and maintaining the program before wrap-up. After program wrap-up, materials and ongoing support were still readily available as the program was integrated into clinic operations.

## Discussion

A conceptual framework was developed from the IAP2 methodology and implementation science structures to guide SE activities in PMP. The conceptual framework proved logical, realistic, and sustainable across participating clinical sites. The actual experiences shared by stakeholders at the clinical sites uniformly aligned with the theoretical constructs in PMP. While the SE approach was guided by the conceptual framework, the strategies used also reflect a real-world application of the PCORI guidelines for SE in breast cancer research [12]. To our knowledge, this is the first publication of a real-world application of a SE approach and relevant guidelines in an EBI to increase mammography adherence among underserved women. Mutual trust was developed first by partnering with BHCTexas who had existing relationships with the identified PMP clinical stakeholders through their clinic membership network. These partnerships extended engagement with stakeholders across the clinic setting: *Decision-Makers*, *Program Champions*, and *Patient Navigators* at FQHCs and charity clinics [1]. Through the existing partnership with BHCTexas, multiple communication modalities were used to explain PMP objectives and gain buy-in from PMP clinical stakeholders. Initial emails were sent to *Decision-Makers* to invite clinics to participate, a webinar was developed to explain the PMP, and adoption meetings were scheduled to obtain agreement to participate. As a part of the agreement, a Memorandum of Understanding (MOU) was a practical tool developed to outline roles and expectations of the clinical stakeholders, BHCTexas, and researchers throughout implementation. The clinical stakeholders provided feedback on PMP first in the clinical site assessment meeting and throughout the stakeholder committee meetings. The clinical site assessment provided stakeholders an opportunity to provide PMP “fit” with current clinic processes during real-time and normal clinic workflow. In addition, practical tools (e.g., Clinic Handbook) were developed by researchers and BHCTexas with feedback from clinical stakeholders. Stakeholders were able to make adaptions to the program materials to synchronize the clinics’ current processes and reflect populations served while ensuring fidelity of the EBI components. Practical tools have been developed in previous research [13, 14], but this paper addresses an ongoing gap in how best to engage stakeholders in adapting practical tools and providing training for effective dissemination of these tools [1]. Stakeholders were provided paper and digital copies of the adapted program materials in the trainings for *Patient Navigators*. The enhanced training for *Patient Navigators* included assigning continuing education unit (CEU) credits through the researchers’ academic institution. Stakeholders were also able to provide feedback on barriers and challenges to PMP implementation throughout stakeholder committee meeting discussion and subsequent onsite clinic support from BHCTexas CHWs. The clinical sites appreciated the ongoing support through PMP implementation to further adapt the program for their specific clinic needs and patient populations while providing sustainable practical tools and intervention activities after program completion.

Stakeholders were engaged in later research stages of implementation and dissemination rather than just in problem identification and intervention planning [1]. Future research might focus on engagement of multiple clinical stakeholders throughout the research process to develop practical tools and trainings. Researchers can build on the SE conceptual framework and strategies described in this paper to ensure intervention adoption, implementation effectiveness, and sustainment of intervention activities. While patients were involved in the EBI development, patients seeking a mammogram screening at the participating FQHC and charity clinics were not involved in PMP implementation. Future research might focus on if and how to include patients in SE across IAP2 phases to better integrate patients throughout the research process. Research in a clinical setting resulted in challenges and limitations. First, due to a shift in our internal record keeping systems, data from all the stakeholder committee meetings conducted were not available for the document review. While this is a limitation of the study, it does not diminish the results derived as the process was replicated for each group of clinics during the project and the interactions with the clinics and the clinic staff were consistent between the groups. Second, midway through the stakeholder committee meeting process, the researchers shifted from collective stakeholder committee meetings to one-on-one meetings with each clinic. It became apparent that clinic staff viewed the staff from other clinics participating in the program as competitors. The adjustment was necessary for researchers to successfully engage all clinical staff during the stakeholder process in a meaningful way.

## Conclusion

PMP is an innovative adaptation of a mammography screening EBI with multi-level SE. SE strategy successes and challenges were identified in adoption, implementation, and sustainment. The overall effectiveness of SE could be replicated when coupled with similar EBIs. Utilizing a theoretical base allowed for effectively bridging the research-practice gap. The conceptual framework can be easily adopted to mammography screening initiatives, or modified to address other community-based cancer screenings programs.

## Data Availability

Not applicable
